# Spatial and economic quantification of provisioning service by eelgrass beds in Lake Notoro, Hokkaido, Japan

**DOI:** 10.1038/s41598-024-54348-7

**Published:** 2024-02-14

**Authors:** Keizo Ito, Shiori Sonoki, Kenji Minami, Susumu Chiba, Hokuto Shirakawa, Toshifumi Kawajiri, Yanhui Zhu, Kazushi Miyashita

**Affiliations:** 1 Graduate School of Environmental Science, Hokkaido University, 20-5 Benten-cho, Hakodate, Hokkaido 040-0051 Japan; 2 Kushiro Fisheries Research Institute, Hokkaido Research Organization, 2-6 Hama-cho, Kushiro, Hokkaido 085-0024 Japan; 3 Field Science Center for Northern Biosphere, Hokkaido University, 20-5 Benten-cho, Hakodate, Hokkaido 040-0051 Japan; 4 Faculty of Bioindustry, Tokyo University of Agriculture, 196 Yasaka, Abashiri, Hokkaido 099-2493 Japan; 5 Fisheries Research Institute, Japan Fisheries Research and Education Agency, 1-5939-22, Suido-cho, Chuo-ku, Niigata, Niigata 951-8121 Japan; 6 Nishi-Abashiri Fishermen’s Association, 1-7-1, Omagari, Abashiri, Hokkaido 093-0045 Japan

**Keywords:** Environmental sciences, Marine biology, Ecosystem services

## Abstract

Eelgrass beds provide a habitat for many high-value fishery resources, and provisioning services, one of the ecosystem services, need to be quantified. However, few examples have been evaluated spatially. We determined the distribution of eelgrass beds in Lake Notoro, a marine lagoon in Hokkaido, Japan, and quantified the provisioning services by the eelgrass beds in relation to *Pandalus latirostris*, a fishery resource. Acoustic measurement surveys of the eelgrass beds and catch surveys of the shrimp were conducted in July and August 2015. The relationship between catch per unit effort (CPUE) of shrimp and the distribution of eelgrass beds was shown. The estimated distribution area of eelgrass beds was 7.07 km^2^. Shrimp was frequently caught at water depths of 3–5 m, approximately 200 m from the edge of the eelgrass beds. The expected catch of shrimp in the fishing area of Lake Notoro in 2015 was 25.37 tons and US$ 463.6 thousand. Eelgrass beds were found to affect the fisheries production not only on the inside but also at the edge and outside. The entire coastal space should be evaluated, while considering the effect of the distribution of eelgrass beds, to quantify the provisioning services.

## Introduction

In the Millennium Ecosystem Assessment, ecosystem services are defined as the benefits that humans receive from ecosystems^1^. Ecosystem services are divided into four categories based on their roles: provisioning, regulating, supporting, and cultural services^1^. Ecosystem services are often expressed their economic value in monetary units^2^. The economic valuation of ecosystem services is important because placing an economic value on nature makes it easier for public and policy makers to recognize its importance^2–4^. The value of ecosystem services on Earth is US$ 33.2680 trillion per year, of which US$ 20.9490 trillion per year is the value of ecosystem services in marine areas^5^. Marine areas are divided into open ocean and coastal zones. The value of coastal zones is US$ 12.5680 trillion per year though their area is one-tenth that of the open ocean^5^. The coastal zones are divided into four biomes, of which the value of the ecosystem services of seagrass and algae beds is high at US$ 3.8010 trillion, although the area occupied by seagrass and algal beds is 6.4% of the coastal zones^5^.

Seagrasses are marine flowering plants that inhabit shallow coastal zones on all continents, except Antarctica, and form extensive meadows^6,7^. The ecosystem services provided by seagrasses were reviewed by Nordlund et al.^8^ and range from providing food for humans and habitats to providing food for aquatic organisms, carbon sequestration, coastal protection, and primary production. Seagrass beds provide habitat and food for aquatic organisms, serving not only as nurseries for juveniles of commercially exploited species but also as fishing grounds for adults^9^. Seagrass beds contribute to fishing production^10^, and the economic valuation of provisioning services, which are ecosystem services, is important. Seagrass beds have been reported to influence the density and biomass of fish populations due to their complex canopy structure^11^. Studies on the influence of seagrass bed size and density on the provision of ecosystem services are also required^8^. Quantification of the spatial provisioning services by seagrass beds is hence necessary. However, there are few examples of such studies. Eelgrass beds are one of the most widely distributed seagrass beds in most of the world's oceans^6^. Eelgrass beds have many important roles, such as increasing fisheries production, improving water quality, and uptake of carbon and nitrogen^12–15^. Eelgrass beds are distributed in Japan from Hokkaido to the Ryukyu Islands^16^. Eelgrass beds in the coastal zone of Japan have seasonality in biomass and shoot density, with the highest values in spring and summer and the lowest values in fall and winter^16^. The provision of habitat and food for aquatic organisms by eelgrass beds have been reported in several studies in Japan^17,18^. Thus, it is important to quantify the spatial and economic provisioning of services during the thriving season, when they are distributed over a wide area.

Lake Notoro, located in Abashiri City in eastern Hokkaido, Japan, is a marine lagoon where eelgrass beds are formed by *Zostera caespitosa*, *Zostera marina*, and *Zostera japonica*^16^. *Z*. *japonica* is found at depths shallower than 1 m, whereas *Z*. *caespitosa* and *Z*. *marina* are found at depths as low as 10 m^16^. The eelgrass beds in Lake Notoro provide habitats for fishery species such as fish, shrimp, and sea urchins, and provide provisioning services^18^. Among them, *Pandalus latirostris* is one of the most valuable fishery resources in Lake Notoro^19^, with an average value of US$ 24.67 kg^−1^ caught in 10 years, excluding the closed season from 2012 to 2021, assuming that 1 US$ is 130 Japanese yen^20^. Shrimp have utilized eelgrass beds throughout their life history^21^. The shrimp fishery in Lake Notoro is limited to summer and the shrimps are caught in shrimp cages. Shrimp cages are used to selectively catch only large female shrimps by limiting their mesh size^19,22^. The Nishi-Abashiri Fishermen’s Association, which oversees the fisheries in Lake Notoro, has been implementing positive management measures for shrimp resource conservation^23^. However, with a decline in eelgrass beds in recent years, the amount of shrimp caught in Lake Notoro has also been declining since its peak of 74 tons in 2000^20^. The relationship between the density of eelgrass beds and that of shrimp was positive in the yearling group but not in the 2 years group which becomes a fishery resource^24^. We can contribute to the sustainable development of fisheries and local communities in Lake Notoro by identifying where shrimp are most likely to be caught in eelgrass beds. From the above, demonstration of the spatial and economic value of provisioning services using eelgrass beds in Lake Notoro is necessary. However, no such studies have been conducted so far.

Acoustic measurement methods are effective in quantifying the spatial distribution of eelgrass beds^25^. These methods can continuously measure the presence and depth of objects in the water by emitting ultrasonic waves into the water and receiving the reflected waves that bounce back from the objects^26^. Acoustic measurement methods can provide information on a wide range of distribution more easily and quickly than direct observation methods^27^. These methods are also less affected by water clarity, and more effective than optimal remote sensing with aircraft, satellites, and unmanned aerial vehicles (UAVs) in determining the spatial structure of seagrass beds in coastal waters^28^. The quantitative mapping of eelgrass beds using acoustic measurement methods has been actively pursued^29–32^. Spatial seagrass structure and depth have been found to affect fish abundance in small scales^33^. Accordingly, potential catch of fishery resources by eelgrass beds can be visualized for the entire coastal area by combining the spatial distribution of eelgrass beds over a wide area obtained by acoustic measurement methods and the relationship between eelgrass beds and fishery resources. This allows a spatial and economic evaluation of the provisioning services by eelgrass beds over a wide area, and has a significant impact on the management of eelgrass beds and fisheries.

In this study, we aimed to spatially and economically evaluate eelgrass beds by quantifying their provisioning services using the relationship between eelgrass beds and *P*. *latirostris* in Lake Notoro as a model case. Three tasks were performed to achieve these goals. First, we mapped the thickness of the eelgrass beds and estimated their distribution areas using acoustic measurement methods. Second, we examined the shrimp catch using shrimp cages and tested a generalized additive model (GAM) for the characteristics of the fishery grounds where *P*. *latirostris* is frequently caught, based on the distribution of eelgrass beds and CPUE of the shrimp. Finally, we mapped the potential CPUE of shrimp using the above model, calculated the potential catch of shrimp, and evaluated the provisioning services provided by eelgrass beds spatially and economically in Lake Notoro.

## Materials and methods

### Field surveys

**Figure 1:**
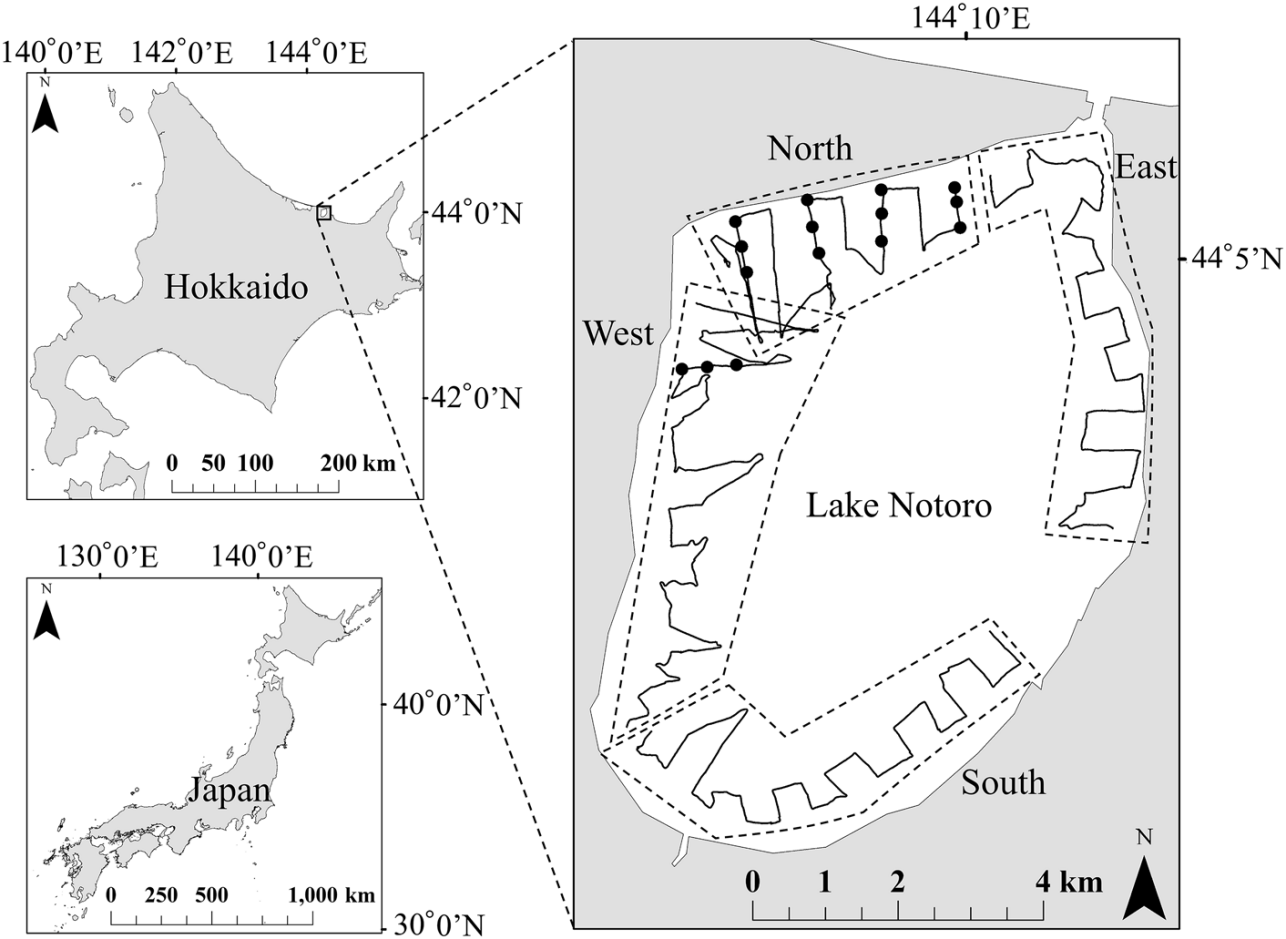
The study area, Lake Notoro, in eastern Hokkaido, Japan. The solid lines show the acoustic survey transects. The closed circles show the points of the catch surveys of *P*. *latirostris*. The map was generated by ArcGIS 10.8 (https://www.esri.com) and does not require any permission from anywhere.

In July and August, when the eelgrass beds were in full bloom, acoustic measurement surveys of the eelgrass beds and catch surveys of the shrimp were conducted (Fig. 1). Acoustic measurement surveys of line transects in Lake Notoro were conducted from July 16 to 17, 2015, to estimate the distribution area of the eelgrass beds using a quantitative echo sounder, KCE-300, with a split beam transducer (120 kHz, Sonic Co., Japan, Table 1). Quantitative echo sounders can assess biomass quantitatively and are different from typical commercial fisheries echo sounders^34,35^. Quantitative echo sounders are also applied to eelgrass beds^32^. We followed a previous study and adopted a high-frequency transducer to prevent resonance by small gases in the seawater^32^. The eelgrass beds in Lake Notoro inhabit water depths up to 10 m^16^. In addition, *Z*. *japonica* is a small eelgrass found at depths shallower than 1 m and is not distributed within the area navigable by ships in Lake Notoro. Therefore, *Z*. *caespitosa* and *Z*. *marina* were selected as target species for our study. The survey area was 16.24 km^2^ on the shoreside, up to an area navigable by ship, and on the offshore side, up to a water depth of 10 m (Fig. 1). The transducer of the quantitative echo sounder was attached to the outside of the ship, such that the surface of the transducer was at a depth of 50 cm from the sea surface to avoid the influence of bubbles. Acoustic measurements were made by navigating the ship at 3 knots on the survey transect lines set at 500 m intervals. The bathymetry of the survey area was estimated from acoustic data measured by kriging^36^, a spatial interpolation method.

**Table 1:** Settings of quantitative echo sounder, KCE-300 with T-182 transducer (Sonic Co.).

KCE-300 with T-182 transducer	
Frequency (kHz)	120
Pulse length (ms)	0.6
Beam width (°)	8.5
Resolution (cm)	3.5
Ping rate (s^−1^)	5
Beam type	Split beam
Weight of transducer (kg)	8.0
Diameter of transducer (cm)	13.0

Catch survey of the shrimp was conducted on August 8–11, 2015, under the same conditions as the catch method used in the fishery of Lake Notoro, and CPUE of the shrimp was calculated. Shrimp cages were conical with a base diameter of 70 cm, height of 30 cm, mesh size of 1 cm, and two entrances with a diameter of 5 cm. Approximately 500 g of frozen Pacific saury bait per cage was folded in half, which is commonly used in shrimp fisheries. The survey was conducted on the same timescale as that of normal fisheries, with cages placed at 4:00 am and retrieved at 4:00 am the next day. The northwest area of Lake Notoro is one of the common shrimp-fishing grounds. Acoustic measurement surveys in July were also found that the eelgrass beds were widely distributed in the northwest, while they were less distributed toward east, with some areas having no eelgrass beds. Thus, the cages were placed northwest to show relationship between distribution of the eelgrass beds and CPUE of the shrimp. The cages were placed at intervals of approximately 350 m, with a fixed line set perpendicular to the nearest shoreline on the line of the acoustic measurement survey of the eelgrass beds. Five lines were prepared with three cages per fixed line, and 15 cages were placed per day for four consecutive days (Fig. 1). The cages were retrieved, the caught shrimps were transferred to a fish container prepared onboard, measured on a platform scale. The caught shrimps were quickly released at the same points where the cages were retrieved.

### Data analysis

**Figure 2:**
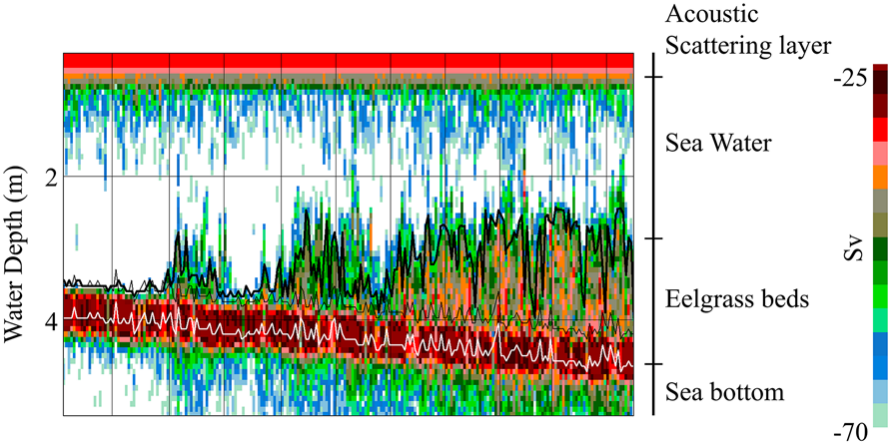
Representative echogram of an eelgrass bed. The white line is the maximum acoustic reflection intensity, the thin black line is raised 45 cm from the white line to account for the influence of a dead zone near the sea bottom, and the thick black line is the upper threshold of the eelgrass beds.

Acoustic data from the quantitative echo sounder were analyzed using Echoview 11.1 (Echoview Software Pty Ltd., Australia). In eelgrass beds, the acoustic backscattering strength can identify seawater, eelgrass beds, and the sea bottom (Fig. 2). The boundary of the surface acoustic scattering layer^37^ was excluded from the analysis according to a previous study^32^. The layer with the strongest acoustic backscattering strength was defined as the lower edge, the boundary between the sea bottom and seagrass beds, and no eelgrass beds less than 45 cm below the sea bottom were considered^32^, to account for the influence of a dead zone near the sea bottom^38^. A histogram of the reflection intensity was obtained to extract the eelgrass beds from the echogram, which was created from the reflection intensity every 2 dB over a 20 m horizontal range, excluding the acoustic scattering layer and the dead zone, and was bimodal: seawater and eelgrass beds^32^. The presence of eelgrass beds can be determined using the mean of the lowest frequency as the boundary between the two modes^32^. In this study, this mean value was calculated at five random sites where eelgrass beds were present and was defined as the threshold for the upper edge of the eelgrass beds. The thickness of the eelgrass beds was defined as the length from the upper edge, defined as the threshold, to the lower edge and the boundary between the sea bottom and eelgrass beds. The mean threshold of reflection intensity between the eelgrass beds and seawater was set at − 53.0 dB.

Acoustic data were extracted at 2 m horizontal distance intervals. The presence or absence and thickness of the eelgrass beds were mapped using ArcGIS 10.8 (ESRI). We estimated the distribution of eelgrass beds between line transects using kriging^36^, a spatial interpolation method for obtaining a value at a point without direct observation from neighboring observations using spatial autocovariance. Because the environment differs among areas, even within the same lake, in this study, the study area was divided into four areas (north, south, east, and west), and kriging was conducted with the parameters set for each area. The presence or absence of eelgrass beds was estimated using indicator kriging, which was used to provide a probability map from a binary function^39^. Next, the thickness of the eelgrass beds in the study area was estimated using ordinary kriging to consider the internal structure of the eelgrass beds, and their distribution area was calculated. Ordinary kriging predicts the value of an unsampled point by a linear combination of neighboring observations^40^.

The CPUE of shrimp, calculated from the catch survey, was defined as the mean of the four days of the CPUE for each site. GAM was used to determine the relationship between the CPUE of the shrimp and eelgrass beds. The response variable was the shrimp CPUE, and the explanatory variables were thickness of the eelgrass beds, patch length of the eelgrass beds, water depth, and distance from the edge of the eelgrass beds obtained from the acoustic data. Acoustic data for the explanatory variables were used in the analysis by dividing the acoustic data by 10 m to represent the eelgrass beds in a detailed range while preserving the characteristics of the patches. A patch of eelgrass beds was defined as one continuously measured eelgrass beds community, and the patch length was the mean of the lengths of each patch. Distance from the edge of the eelgrass beds was defined as the distance from each site in the catch survey to the nearest eelgrass bed edge-derived kriging, with a positive value inside the eelgrass bed and a negative value outside. Analysis was performed using the ‘mgcv’ package of the free R^41^, and the best model was determined based on Akaike’s Information Criterion (AIC).

### Quantification of the provisioning services

The best model in the AIC and acoustic data from the eelgrass beds in July 2015 were used to estimate the potential CPUE of shrimp in Lake Notoro. Acoustic data were separated every 10 m and the thickness of the eelgrass beds, patch length of the eelgrass beds, water depth, and distance from the edge of the eelgrass beds were extracted to use spatial analysis. The location information of the data within 10 m of the acoustic data was averaged and used as the representative location information for the point. The shrimp CPUE at each site was estimated using the best model. The potential CPUE of the shrimp was mapped every 0.5 kg cage^−1^ from the CPUE of each point using ordinary kriging. Fishermen in Lake Notoro often use water depths of 3–5 m as shrimp fishing grounds. In recent years, the eastern area of Lake Notoro has been set as a prohibited fishing area because of a decline in shrimp catch. Therefore, areas with water depths of 3–5 m, excluding the eastern area, were extracted as actual fishing areas from the potential CPUE map. Potential CPUE in the actual fishing areas was calculated by multiplying the estimated CPUE by their respective areas, then summing them and dividing by the total area of the actual fishing areas. This potential CPUE was multiplied by the actual number of fishermen, the number of shrimp cages per fisherman per day, and the mean number of fishing days in the past 3 years to obtain the expected catch of shrimp in Lake Notoro in 2015. To quantify the provisioning service of the eelgrass beds, the expected catch of shrimp was estimated by multiplying the catch by the amount per kilogram. The amount per kilogram of shrimp was calculated using the mean of the amounts for 10 years prior to 2014 (2375 Japanese yen kg^−1^) when 1 US$ was 130 Japanese yen^20^.

## Results

### Distribution area of the eelgrass beds

**Figure 3:**
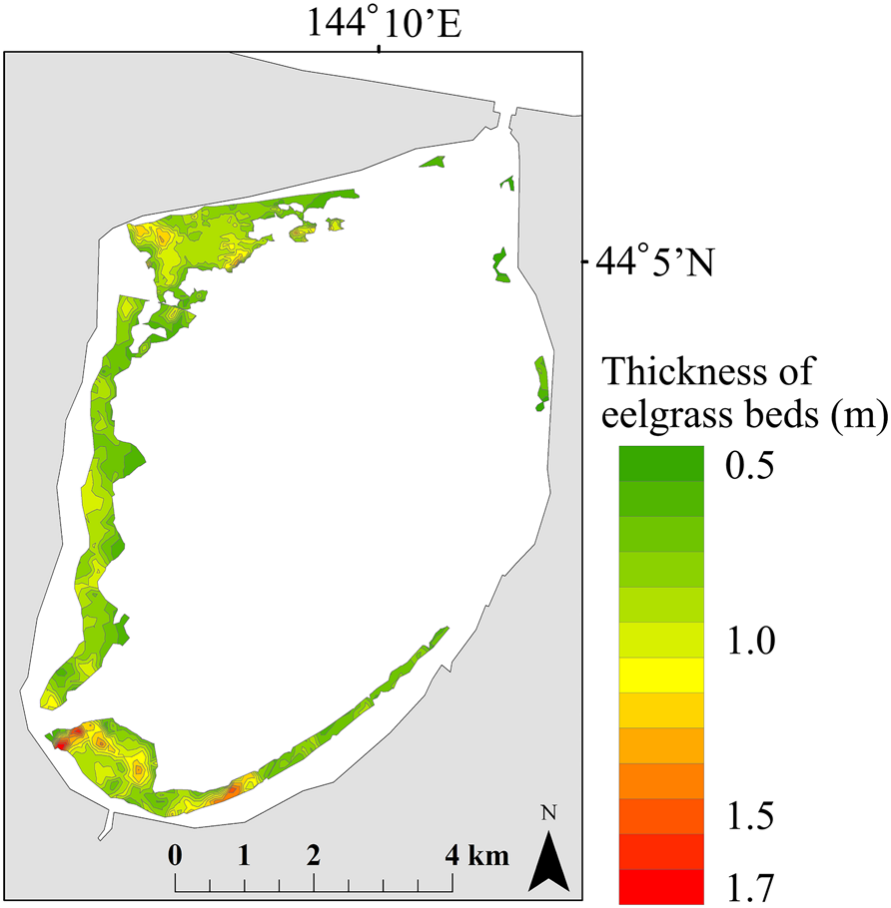
Estimated distribution area of eelgrass beds in July 2015. The map was generated by ArcGIS 10.8 (https://www.esri.com) and does not require any permission from anywhere.

The thickness of eelgrass beds in Lake Notoro in July 2015 was 0.76 ± 0.12 m. The total distribution area of the eelgrass beds estimated by kriging was 7.07 km^2^ and they were present in 43.53% of the survey area (Fig. 3). No eelgrass beds were observed at depths greater than 6 m in the estimated distribution. By sea area, the largest distribution area was 2.78 km^2^ in the west, 2.13 km^2^ in the south, 1.97 km^2^ in the north, and 0.18 km^2^ in the east (Fig. 3).

### Spatial relationship between the CPUE of *P. latirostris* and eelgrass beds

**Figure 4:**
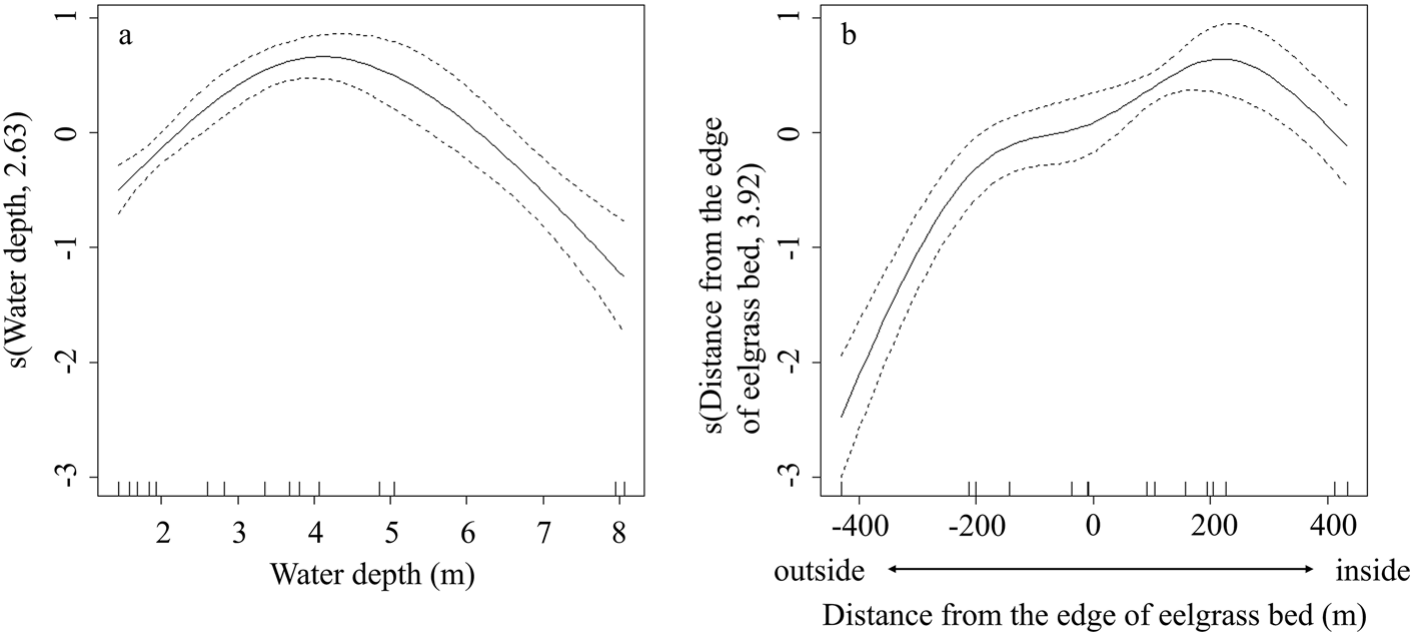
GAM-predicted smooth splines of the response variable CPUE of* P*. *latirostris* as a function of the explanatory variables (**a**) water depth and (**b**) distance from the edge of the eelgrass bed. The tick marks inside the x-axis are observed data points of each variable. The y-axis represents the partial effect of each variable. The degrees of freedom for non-linear fits are in parenthesis on the y-axis. The dotted lines represent the 95% confidence intervals of the smooth spline functions.

The mean CPUE of the shrimp cages at each of the 15 sites in August ranged from 0.07 to 3.10 kg (Table 2). The GAM results showed that the model with the smallest AIC and the best model included the thickness of the eelgrass beds, patch length of the eelgrass beds, water depth, and distance from the edge of the eelgrass beds (Table 3). The catch of the shrimp was low on the shallow shoreside where the eelgrass beds were densely distributed, increased as it approached the edge, and decreased as it moved away from the margin and the water depth became deeper. In particular, shrimp were most likely to be caught at depths of 3–5 m, approximately 200 m from the edge of the eelgrass beds (Fig. 4).

**Table 2:** CPUE of *P*. *latirostris* from the catch survey for 4 days in August 2015.

Point	Latitude	Longitude	CPUE (kg cage^−1^)				Mean (kg cage^−1^)
			8-Aug	9-Aug	10-Aug	11-Aug	
A1	44.0727	144.1151	1.26	1.95	1.48	1.42	1.53
A2	44.0728	144.1194	1.50	2.55	2.84	2.66	2.39
A3	44.0729	144.1244	0.44	0.10	0.74	0.86	0.54
B1	44.0906	144.1255	0.06	0.70	1.22	0.32	0.58
B2	44.0875	144.1264	2.20	1.30	2.40	2.46	2.09
B3	44.0843	144.1270	2.90	2.24	2.80	2.78	2.68
C1	44.0929	144.1380	0.90	1.30	1.10	0.77	1.02
C2	44.0895	144.1386	2.40	2.30	1.84	2.64	2.30
C3	44.0862	144.1395	5.00	4.14	2.50	0.74	3.10
D1	44.0937	144.1507	1.70	2.40	1.34	1.04	1.62
D2	44.0907	144.1506	2.10	2.00	4.00	4.54	3.16
D3	44.0873	144.1503	4.00	2.40	2.50	1.16	2.52
E1	44.0935	144.1633	0.50	0.64	1.06	1.54	0.94
E2	44.0917	144.1635	2.26	3.90	2.98	1.26	2.60
E3	44.0885	144.1639	0.08	0.14	0.02	0.04	0.07

**Table 3:** Summary results for the parametric coefficients and smooth terms of the final GAM selected to model the CPUE of* P*. *latirostris*.

Family	Gamma			
Link function	log			
Adjusted R-squared	0.92			
Deviance explained (%)	98.3			

Parametric coefficients	Estimate	Std. Error	*t* value	Pr( >|t|)
Intercept	1.01	0.25	4.06	0.0082
Thickness of eelgrass bed	− 1.03	0.39	− 2.68	0.0400
Distance of eelgrass bed	− 0.03	0.03	− 1.13	0.3067

Smooth terms	edf	Ref.df	F	*p* value
s(Water depth)	2.63	3.08	18.05	0.0044
s(Distance from the edge of eelgrass bed)	3.92	3.99	27.78	0.0012

### Calculation of the provisioning services by the eelgrass beds

**Figure 5:**
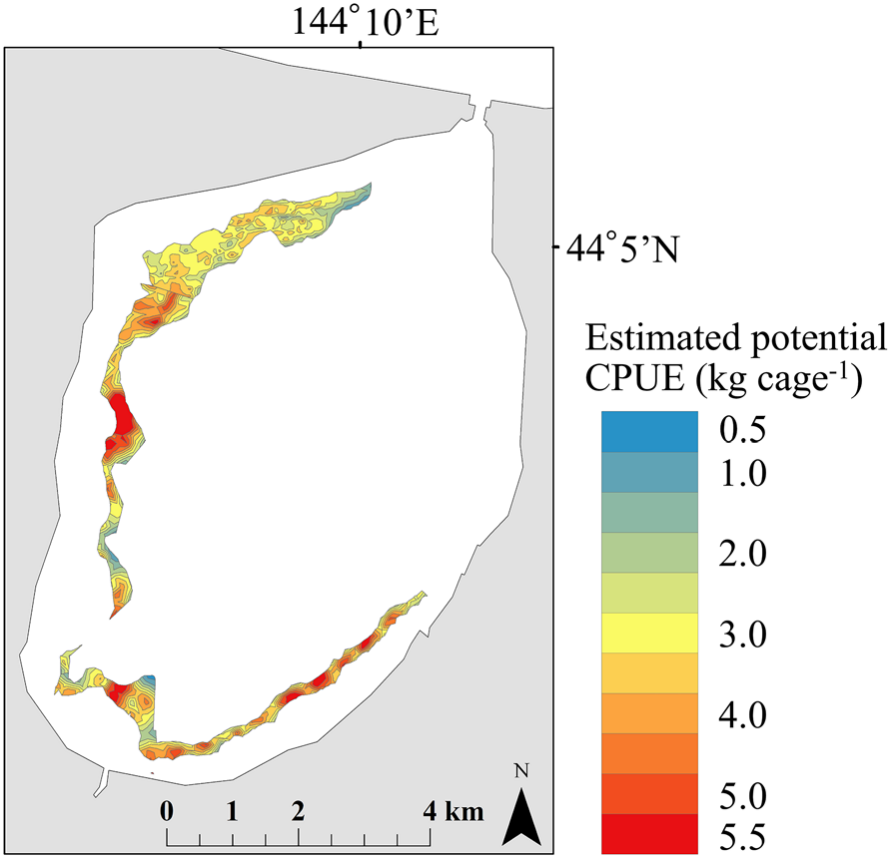
Map of the estimated potential CPUE of* P*. *latirostris* obtained by extracting a range of 3–5 m in depth, to be used in the actual fishery. The map was generated by ArcGIS 10.8 (https://www.esri.com) and does not require any permission from anywhere.

From the potential map spatially interpolated by kriging using the best model, the distribution area of shrimp was 4.95 km^2^ in the actual fishing areas at water depths of 3–5 m, excluding a prohibited fishing area (Fig. 5). The potential CPUE, obtained by multiplying the estimated CPUE by their respective areas, then summing them and dividing by the total area of the actual fishing areas, was calculated to be 3.11 kg cage^−1^. Multiplying the CPUE per unit area by the actual number of fishermen (32), the number of shrimp cages per fisherman per day (15), and the mean number of fishing days in the past 3 years (17), the expected catch of shrimp in Lake Notoro was calculated to be 25.37 tons. Multiplying the catch by the amount per kilogram of shrimp, the expected catch of shrimp in Lake Notoro in 2015 was calculated to be 60.26 million Japanese yen. At an exchange rate of 130 Japanese yen to US$, the catch was valued at US$ 463.6 thousand.

## Discussion

The distribution area of eelgrass beds in July 2015, obtained using a quantitative echo sounder was 7.07 km^2^, and no eelgrass beds were identified at depths greater than 6 m. A spot survey by scuba diving in July 1996 revealed that the eelgrass beds were mainly distributed at depths of 0.6–6.5 m^42^. The distribution area of eelgrass beds in Lake Notoro was estimated to be 10.05 km^2^ based on a survey conducted in July 1996^42^; compared to this study, eelgrass beds have decreased over the last 19 years to 2015, although the measurement methods differ. Results from June 2013 at the same site as the 1996 survey reported that eelgrass beds were not identified at depths greater than 4 m and that the biomass of the eelgrass beds had decreased compared to that in 1996^18^. In particular, at the three sites surveyed in the eastern area, the eelgrass beds found at all the points in 1996 were not found in 2013^18^. In this study, the area of eelgrass beds in the eastern was also 0.18 km^2^, almost undistributed. The increase in the number of days with strong winds from the northwest in spring may be one of the reasons^18^. The surface of Lake Notoro is covered with sea ice during winter and the surface ice thawing in spring causes scouring^43,44^. Strong winds from northwest, which often blow in spring, have increased since 1996^18^, and this study also suggests that they have had a negative impact on the distribution of eelgrass beds in recent years in eastern and some southern areas.

The distribution of eelgrass beds in July 2015 varied by area, with the eastern area having a smaller distribution area than the other areas. The slope of sea bottoms in Lake Notoro is steep on the east side, while it is gentle on the north and west sides. The habitat range of eelgrass beds is narrow on the east side and wide on the north and west sides. Strong slope of sea bottoms causes sand surface fluctuations in eelgrass beds, which can lead to the outflow and burial of seeds and newly recruited plants^45^. On the other hand, eelgrass can be more abundant in gentle terrain than in steep terrain^46^. The strong slope of the eastern part of Lake Notoro is thought to negatively affect growth. In addition to the different areas of the bathymetric zone where eelgrass beds could be distributed, differences in the sea bottom slope also caused differences in the distribution in each area.

The CPUE of the shrimp was related to the distribution of eelgrass beds and water depth and could be modeled. The model was able to reveal where shrimp was likely to be caught based on distance from the edge of the eelgrass beds, spatial structure of the eelgrass beds, and water depth. Shrimps were frequently caught near the edge of the eelgrass beds at water depths of 3–5 m, they were caught less frequently in the eelgrass beds closer to the shore and were absent in the offshore eelgrass beds. In Notsuke Bay, which is located in the eastern part of Hokkaido, similar to Lake Notoro, the relationship between the density of shrimp and that of eelgrass beds is positively correlated in the yearling group, with the shrimp inhabiting high-density eelgrass beds, but there was no correlation in the 2 years group, and the dependence on eelgrass bed density becomes weaker with growth^24^. Shrimp changes the spatial use of eelgrass beds at different growth stages. High-density eelgrass beds provide shelter from predators and are used as hiding sites by younger shrimp groups^24^. In Lake Notoro, the shrimp prefers seagrass beds with high shoot density and tend to prefer *Z*. *caespitosa* to *Z*. *marina*^18^. This suggests that the higher density serves as a shelter and *Z*. *caespitosa* with its canopy structure is preferred^18^. In experiments using artificial seagrass units, mature shrimp has been reported to congregate at the edges of eelgrass patches^47^. In addition, mature shrimp leaves the eelgrass patches at night to utilize unvegetated areas, owing to reduced predation pressure and food requirements^47^. In other words, mature shrimp uses not only the interior of eelgrass patches but also the edges and unvegetated areas of the patches. Offshore eelgrass beds were patchy and discontinuous in distribution. The patchy distribution created a sparse space between the eelgrass beds on the offshore side. In this study, we conducted research under the same conditions as those in an actual fishery. The shrimp cages used can selectively catch large shrimp^19,22^. Therefore, we suggested that the catch of large shrimp was low in the high-density eelgrass beds on the shoreside and high in the eelgrass beds near the edge on the offshore side in the best model. We also suggested that the catch of large shrimp was decreased offshore where there were no eelgrass beds to avoid predators.

Based on the acoustic data of the eelgrass beds measured by the quantitative echo sounder and the best model by the GAM, we estimated the potential CPUE of shrimp and created a potential map of shrimp in Lake Notoro in 2015. Considering the actual number of fishermen, the number of shrimp cages per fisherman per day, and the mean number of fishing days in the past 3 years, we were able to estimate the expected catch (25.37 tons) and catch (US$ 463.6 thousand) of the shrimp in 2015, quantifying the provisioning service of the eelgrass beds. The actual catches of the shrimp from 2012 to 2014 in Lake Notoro were 17.4, 17.1, and 15.4 tons, respectively, and the catches were US$ 283.9, 345.5, and 387.1 thousand, respectively, at 130 Japanese yen to the US$^20^. Fishermen decide where to place shrimp cages based on their own experience; the main fishing grounds are near the edges of the eelgrass beds. In this study, we found that shrimp tended to be caught frequently near the edge of the eelgrass beds but also outside the eelgrass beds. Therefore, the catches estimated in this study were higher than the historical catches. In other lagoons, quantification of provisioning services from the biomass of several commercial fish in vegetated and unvegetated areas of eelgrass beds has been reported^48^. In this study, a significant feature is that the spatial distribution relationship between eelgrass beds and shrimp was shown and the potential map was created to calculate the provisioning services. A spatial assessment of the entire coastal area, including areas where eelgrass beds do not exist, is important for quantifying the provisioning services of eelgrass beds.

In this study, using the eelgrass beds of Lake Notoro and shrimp as a model case, the relationship between the eelgrass beds and shrimp was spatially clarified, and the value of the provisioning services was estimated. This study, which combines an acoustic measurement survey of seagrass beds and a fishing survey of fishery resources associated with seagrass beds, is extremely valuable for fisheries in coastal areas. As mentioned, the distribution area of the eelgrass beds in Lake Notoro decreased over the 19 years from 1996 to 2015. Seagrass beds, including eelgrass beds, have been disappearing globally at a rate of 110 km^2^ per year since 1980^49^. In many areas, the loss of seagrass beds has been reported to decrease fishery production, whereas the recovery of seagrass beds has been reported to increase fishery production^50,51^. The Nishi-Abashiri Fishermen's Association, which oversees the fisheries of Lake Notoro, calculates shrimp abundance using the distribution area of eelgrass beds. However, the area of eelgrass beds was based on the area measured in 1996 by Hokkaido Regional Development Bureau^42^, and in light of the results of this study, the abundance of shrimp may have been overestimated. The shrimp catch has been on a downward trend since 2000^20^. The potential map of CPUE for shrimp spatially presented in this study will be useful for fishermen not only to develop new fishing grounds with fishing potential and improve fishing efficiency but also to conserve the fishing grounds of shrimp and eelgrass beds to recover resources. Eelgrass beds should be continuously monitored for distribution, and ecosystem services should be monitored as the distribution changes. The quantitative echo sounder used in this study can measure a wide area of eelgrass beds in a short period and estimate their horizontal and vertical distributions by extracting the height of the eelgrass beds from the acoustic data. The productivity of an eelgrass population is closely related to its three-dimensional horizontal structure^52^. The complex canopy structure of eelgrass beds provides habitat and food for a wide range of vertebrates and invertebrates^53,54^. Furthermore, eelgrass beds, as in this study, have been shown to affect fishery production outside their habitat. The spatial assessment of eelgrass beds conducted in this study is expected to become more important in the future and will lead to the assessment of various ecosystem services.

## Data Availability

The datasets are available from the corresponding author on reasonable request.
